# Supplementary material for: ELISA-Based Assay for Studying Major and Minor Group Rhinovirus–Receptor Interactions
Source: Vaccines (Basel). 2020 Jun 18;8(2):315. doi: 10.3390/vaccines8020315 (PMC7350259; doi:10.3390/vaccines8020315)
Supplement: Supplementary file 1 [file vaccines-08-00315-s001.zip › Table S1.pdf]

Sample ID	Registered sequence	Total score	Query Cover[1] (%)	E value[2]	Identity (%)	Accession
RV2	Human rhinovirus 2, complete sequence	854	100	0.0	100	X02316.1
RV8	Human rhinovirus 8 strain, ATCC VR-1118 complete genome	785	100	0.0	100	FJ445113.1
RV14	Human rhinovirus type 14 (HRV14), complete genome	821	99	0.0	100	K02121.1
RV16	Human rhinovirus type 16, polyprotein gene, complete CDS	854	100	0.0	99.78	L24917.1
RV18	Human rhinovirus 18 strain, polyprotein gene, partial	717	75	0.0	100	AY016407.1
RV25	Human rhinovirus 25 strain, ATCC VR-1135, complete genome	859	100	0.0	100	FJ445123.1
RV29	Human rhinovirus 29 strain, ATCC VR-1139, complete genome	795	100	0.0	100	FJ445125.1
RV44	Human rhinovirus 44, complete genome	865	100	0.0	100	DQ473499.1
RV54	Human rhinovirus 54 strain, ATCC VR-1164, complete genome	822	100	0.0	100	FJ445138.1
RV72	Human rhinovirus 72 strain, ATCC VR-1182, complete genome	822	100	0.0	100	FJ445153.1
RV89	Human rhinovirus 89 strain, ATCC VR-1199, complete genome	813	100	0.0	100	FJ445184.1

[1]Query cover – an overlap between a searched and a reference sequence, [2]E value – a random background noise.
