# Supplementary figures and images for: ELISA-Based Assay for Studying Major and Minor Group Rhinovirus–Receptor Interactions
Source: Vaccines (Basel). 2020 Jun 18;8(2):315. doi: 10.3390/vaccines8020315 (PMC7350259; doi:10.3390/vaccines8020315)

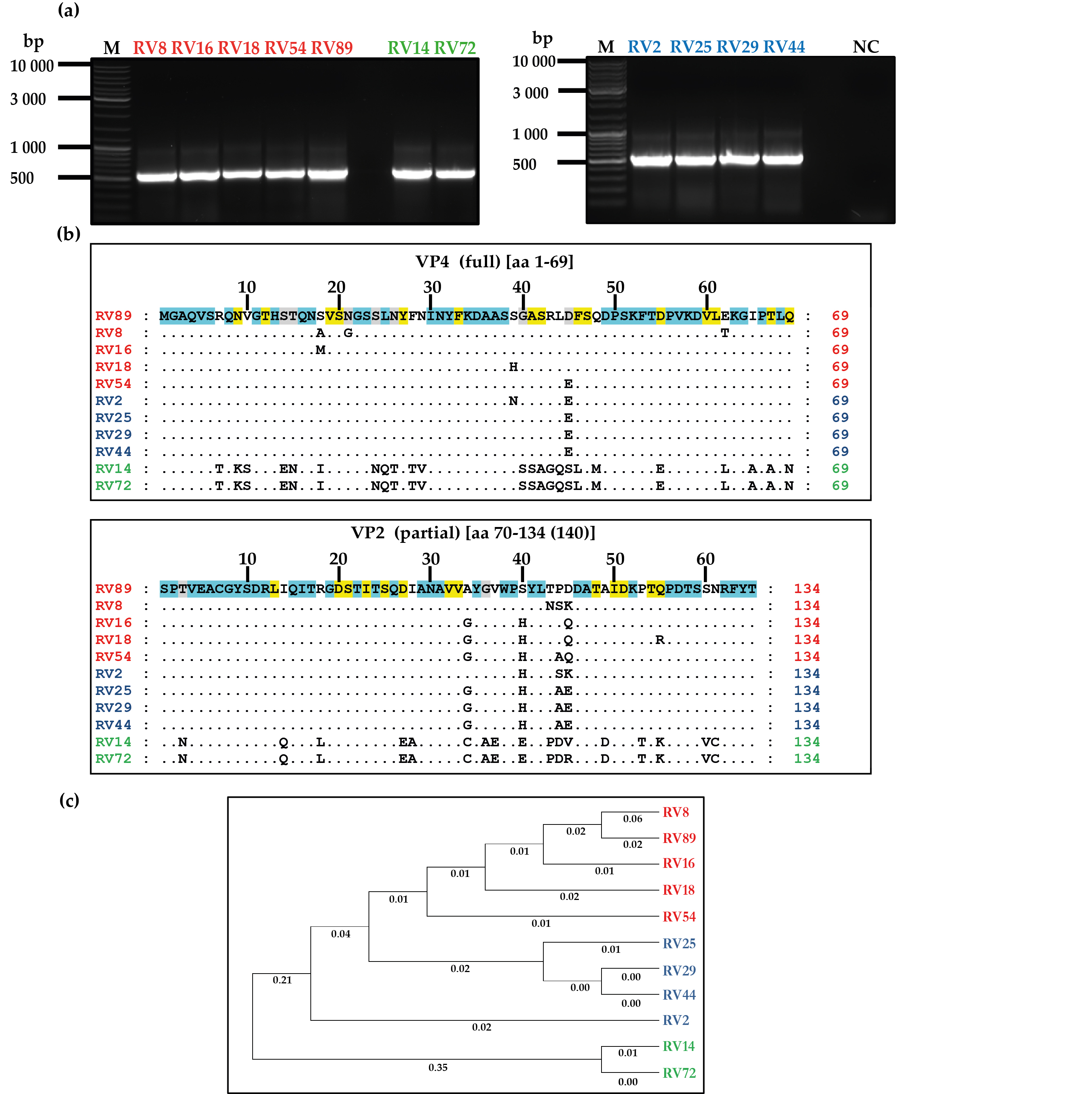

Supplement: Supplementary file 1 [file vaccines-08-00315-s001.zip › Figure S1.jpg]

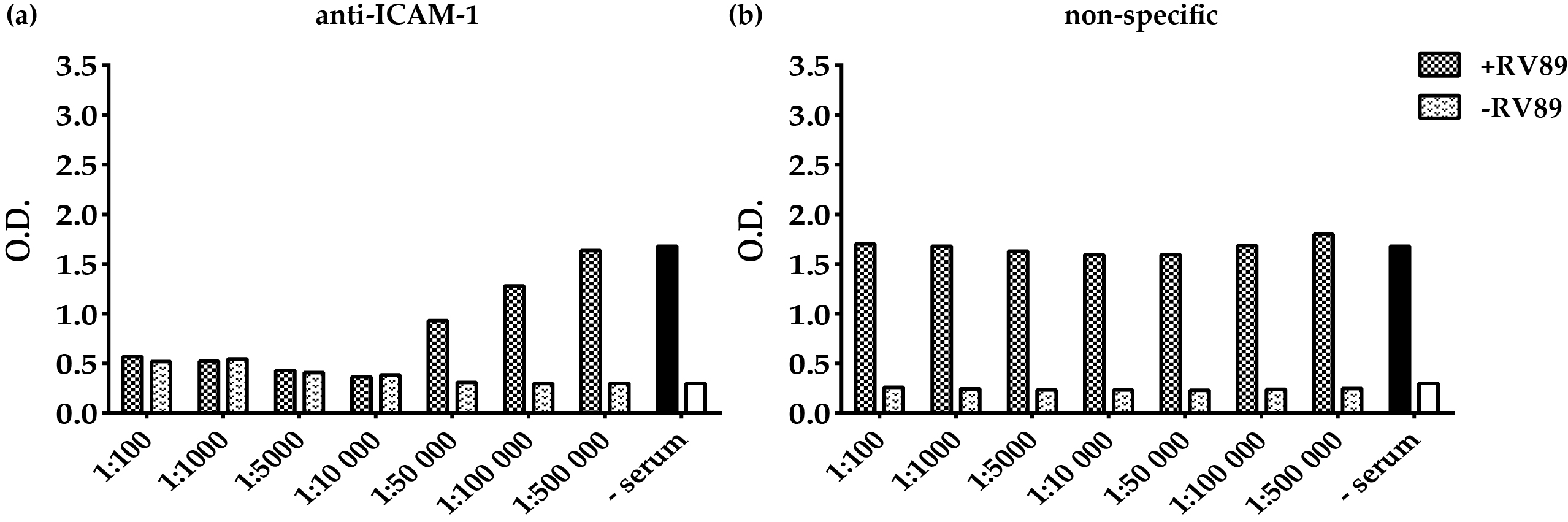

Supplement: Supplementary file 1 [file vaccines-08-00315-s001.zip › Figure S2.jpg]
